# Prognostic value of CA20, a score based on centrosome amplification-associated genes, in breast tumors

**DOI:** 10.1038/s41598-017-00363-w

**Published:** 2017-03-21

**Authors:** Angela Ogden, Padmashree C. G. Rida, Ritu Aneja

**Affiliations:** 1 Department of Biology, Georgia State University, Atlanta, GA USA; 2 Novazoi Theranostics, Inc., Rolling Hills Estates, CA USA

## Abstract

Centrosome amplification (CA) is a hallmark of cancer, observable in ≥75% of breast tumors. CA drives aggressive cellular phenotypes such as chromosomal instability (CIN) and invasiveness. Thus, assessment of CA may offer insights into the prognosis of breast cancer and identify patients who might benefit from centrosome declustering agents. However, it remains unclear whether CA is correlated with clinical outcomes after adjusting for confounding factors. To gain insights, we developed a signature, “CA20”, comprising centrosome structural genes and genes whose dysregulation is implicated in inducing CA. We found that CA20 was a significant independent predictor of worse survival in two large independent datasets after adjusting for potentially confounding factors. In multivariable analyses including both CA20 and CIN25 (a gene expression-based score that correlates with aneuploidy and has prognostic value in many types of cancer), only CA20 was significant, suggesting CA20 captures the risk-predictive information of CIN25 and offers information beyond it. CA20 correlated strongly with CIN25, so a high CA20 score may reflect tumors with high CIN and potentially other aggressive features that may require more aggressive treatment. Finally, we identified processes and pathways differing between CA20-low and high groups that may be valuable therapeutic targets.

## Introduction

CA is a hallmark of cancer observable in ≥75% of breast tumors^1^ that promotes invasive behavior^2^ and enhanced migratory ability^3^ in cancer cells. In addition, the presence of supernumerary centrosomes results in a transient multipolar intermediate in mitosis that promotes merotelic microtubule-kinetochore attachments^4^. To resolve spindle multipolarity and thereby avoid mitotic catastrophe or multipolar mitosis, which could lead to cell death, the cell clusters centrosomes into two polar groups, allowing bipolar division to occur; however, attachment errors persist in the spindle and chromosome missegregation occurs. CIN allows the cell to sample the fitness landscape and acquire a more aggressive karyotype and also promotes intratumor heterogeneity, which fosters chemoresistance^5^. It was recently demonstrated that transient induction of CA in p53-deficient epidermis causes aneuploidy and spontaneous skin cancer development in mice^6^. Given that CA promotes tumorigenesis and aggressive phenotypes and is common among breast tumors, it may have value as a prognostic biomarker in breast cancer and could guide treatment decisions.

Although several groups have performed semi-quantitative assessments of CA in patient tumors using microscopy, few have correlated CA with clinical outcomes, and none of these data are in the public domain. It would thus be valuable to be able to assess CA in publicly available datasets, such as microarray datasets, many of which have clinicopathologic and outcome annotation for breast cancer patients. Our lab previously developed a four-gene signature, which includes two genes for centrosome structural proteins and two genes whose overexpression induces CA, called the Centrosome Amplification Index (CAI), which we found stratifies breast cancer patients into two groups with significantly different overall survival (OS) in Kaplan-Meier analysis^3^. Another group developed a Centrosome Index (CI), comprising four centrosome structural genes, that correlates with CA and is an independent predictor of poor OS in multiple myeloma patients in multivariable analysis^7, 8^. Given that CA can be caused by dysregulation of the expression of many different genes, there is a need to define a more comprehensive gene signature that may be able to identify a greater proportion of tumors with CA, which may arise through a variety of molecular pathways. Thus, in the present study, we define a gene expression signature, “CA20”, that includes 19 genes that have been experimentally demonstrated to induce CA when dysregulated, many of which also have known structural roles in the centrosome (such as SASS6, the primary component of the centriolar cartwheel structure^9^ and CEP152, a key pericentriolar material component^10^, both of which are among the most abundant proteins in the centrosome in several cell lines^11^) (Supplementary Table 1), along with *TUBG1*, which encodes the most abundant centrosomal protein and is primarily responsible for microtubule nucleation, key to centrosomal function^11^. Our objective was to test the prognostic value of CA20 after adjusting for potentially confounding factors in multiple breast cancer cohorts and to explore processes, pathways, and oncogenic signatures that are associated with a high CA20 score. Because CA causes CIN, we were also interested in comparing the prognostic value of CA20 with that of the CIN score “CIN25”, which correlates with total functional aneuploidy and predicts worse outcomes in a variety of cancers^12^, determining which of these two scores has the most significant impact on outcomes when included together in multivariable models of survival, and comparing processes, pathways, and oncogenic signatures that are enriched in tumors with high CA20 and CIN25 scores.

## Results

We tested the ability of CA20 and CIN25 to risk-stratify breast cancer patients in two datasets, the METABRIC and TCGA datasets, comprising n = 1,969 primary breast cancers with breast cancer-specific survival (BCSS) annotation and n = 524 primary invasive breast cancers with OS annotation, respectively. The METABRIC dataset was split into discovery and validation sets. The TCGA dataset was not split because power analysis suggested the subsets would potentially be too small, so bootstrapping was instead used to obtain more reliable estimates of population parameters.

### METABRIC dataset

**Figure 1:**
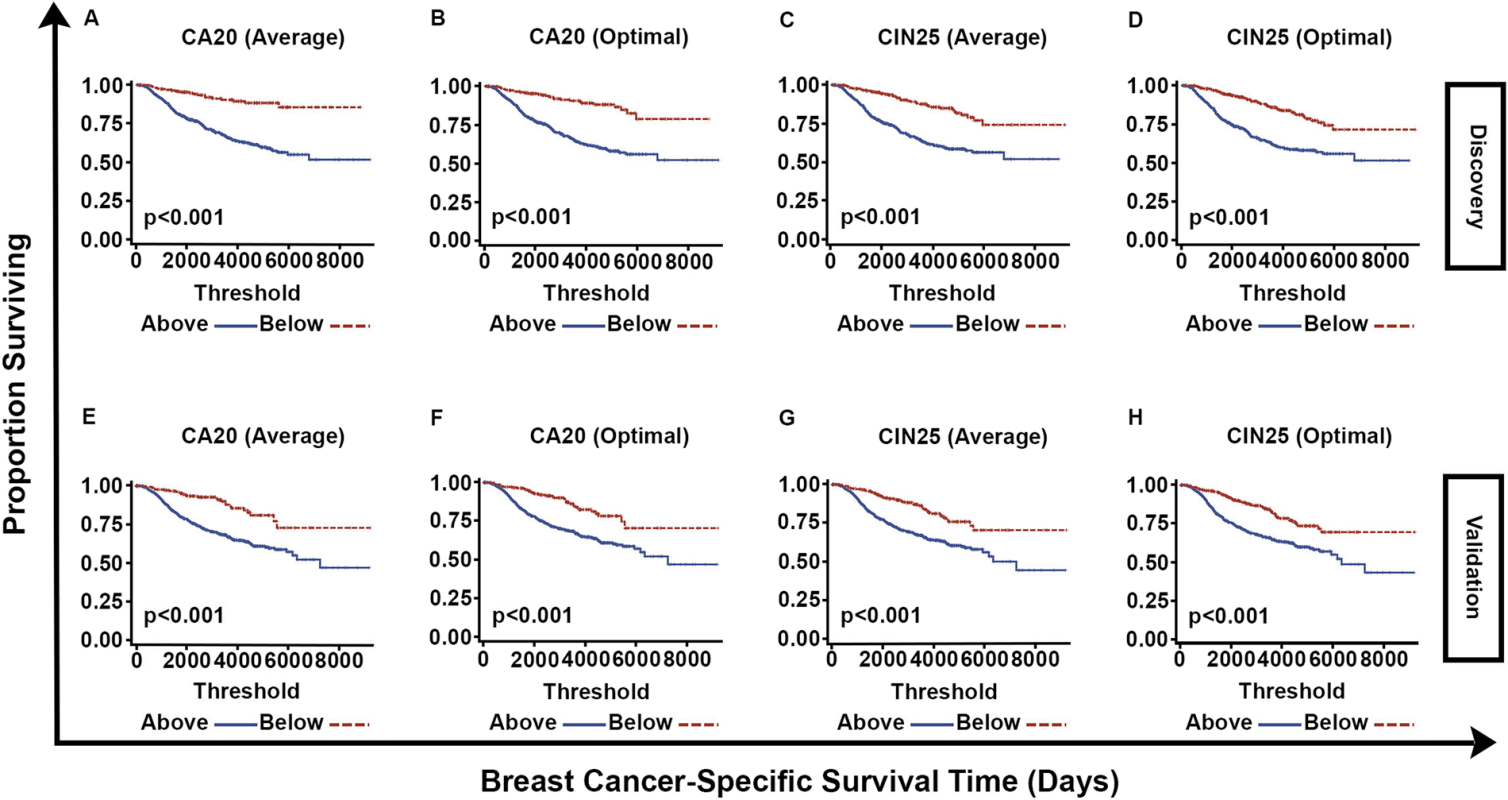
Plots of Kaplan-Meier product limit estimates of breast cancer-specific survival of patients in METABRIC discovery and validation sets stratified by (**A**,**E**) CA20 (average value), (**B**,**F**) CA20 (optimal threshold), (**C**,**G**) CIN25 (average value), and (**D**,**H**) CIN25 (optimal threshold), respectively. Average values and optimal thresholds were determined using the discovery set.

Stratification was conducted according to average CA20 and CIN25 scores found in the discovery set as well as optimal cutpoints in CA20 and CIN25 scores found in the discovery set based on the log-rank test. In Kaplan-Meier plots, stratification into high- and low-BCSS groups based on the average and optimal cutpoints in CA20 and CIN25 scores was significant in both the discovery and validation sets (p < 10^−6^ for all, Fig. 1; see Tables 1 and 2 for descriptive statistics of study datasets). When both CA20 and CIN25 (both stratified by the average score) were entered as covariates in full multivariable models using discovery set data, only CA20 (stratified by the average score) appeared in the final model, and it was a significant predictor of BCSS (Hazard Ratio [HR] = 2.88, p < 0.001; Table 3). In the validation set too, CA20 (stratified by the average score) remained a significant predictor in the final model (HR = 2.13, p﻿ < 0.001). Common significant covariates between discovery and validation set final models included tumor stage and chemotherapy. When both CA20 and CIN25 (both stratified by the optimal cutpoint) were entered as covariates in full multivariable models using discovery set data, both covariates appeared in final models but only CA20 (stratified by the optimal cutpoint) significantly affected BCSS (HR = 2.13, p=0.006;﻿ Table 4). In the validation set, CA20 (stratified by the optimal cutpoint) remained a significant predictor (HR = 1.82, p = 0.028), whereas CIN25 (stratified by the optimal cutpoint) did not significantly impact BCSS. Common significant covariates between discovery and validation final multivariable models of BCSS included tumor stage and chemotherapy, as was found when stratifying by average signature scores. Thus, CA20 (whether stratified by the average score or optimal cutpoint) is a significant predictor of BCSS after adjusting for stage and chemotherapy, whereas CIN25 (whether stratified by the average score or optimal cutpoint) is not an independent predictor in these models.

**Table 1:** Descriptive statistics for the METABRIC breast discovery and validation sets.

Variable	Level (categorical variables) or Statistic (continuous variables)	Discovery	Validation
Nottingham grade	1	80	86
	2	404	363
	3	464	486
	Missing	37	51
Tumor stage	0	230	262
	I	188	174
	II	299	274
	III	45	41
	IV	4	6
	Missing	219	229
Chemotherapy	No	764	790
	Yes	221	196
	Missing	0	0
Hormone therapy	No	378	375
	Yes	607	611
	Missing	0	0
Radiotherapy	No	406	399
	Yes	579	587
	Missing	0	0
Subtype	Luminal	647	623
	TNBC	126	124
	HER2	207	228
	Missing	5	11
CA20 group (average)	Low	234	243
	High	751	743
	Missing	0	0
CIN25 group (average)	Low	337	329
	High	648	657
	Missing	0	0
CA20 group (optimal)	Low	274	278
	High	711	708
	Missing	0	0
CIN25 group (optimal)	Low	405	402
	High	580	584
	Missing	0	0
Age at diagnosis (years)	Mean	60.80	61.50
	Median	61.28	62.56
	Standard Deviation	13.01	12.92
	Minimum	21.93	26.36
	Maximum	92.14	96.29
	Number Missing	0	0
CA20	Mean	30.46	30.33
	Median	30.08	29.86
	Standard Deviation	5.74	5.83
	Minimum	17.27	17.70
	Maximum	49.25	48.45
	Number Missing	0	0
CIN25	Mean	53.30	53.05
	Median	52.84	52.90
	Standard Deviation	13.08	13.45
	Minimum	22.16	20.90
	Maximum	90.67	88.46
	Number Missing	0	0

**Table 2:** Descriptive statistics for the TCGA breast dataset.

Variable	Level (categorical variables) or Statistic (continuous variables)	Value
AJCC stage	I	49
	II	241
	III	92
	IV	13
	Missing	129
CA20 group (average)	Low	240
	High	284
	Missing	0
CA20 group (optimal)	Low	238
	High	286
	Missing	0
CIN25 group (average)	Low	256
	High	268
	Missing	0
CIN25 group (optimal)	Low	514
	High	10
	Missing	0
Age at diagnosis (years)	Mean	58.11
	Median	59.00
	Standard Deviation	13.19
	Minimum	26.00
	Maximum	90.00
	Number missing	74
CA20 score	Mean	23.66
	Median	24.57
	Standard Deviation	8.91
	Minimum	0.00
	Maximum	45.70
	Number missing	0
CIN25 score	Mean	45.37
	Median	46.07
	Standard Deviation	16.71
	Minimum	0.00
	Maximum	88.52
	Number missing	0

**Table 3:** Final multivariable Cox proportional-hazards models of breast cancer-specific survival including CA20 and CIN25 (stratified by average scores) in full models using METABRIC data.

Covariates	Discovery set				Validation set			
	p-value	HR	95% CI for HR		p-value	HR	95% CI for HR	
			Lower	Upper			Lower	Upper
CA20 (high)	<0.001	2.88	1.67	4.96	<0.001	2.38	1.50	3.79
Stage 0	<0.001				<0.001			
Stage I	0.002	0.49	0.31	0.76	0.002	0.49	0.31	0.77
Stage II	0.002	0.58	0.41	0.82	0.85	0.97	0.69	1.36
Stage III/IV	0.001	2.28	1.39	3.74	0.001	2.23	1.37	3.62
Chemotherapy	<0.001	2.09	1.42	3.06	0.008	1.64	1.14	2.36
Hormone therapy	0.031	1.43	1.03	1.98	0.84	0.97	0.71	1.33
Radiotherapy	0.082	0.75	0.54	1.04	0.59	1.09	0.80	1.48
Subtype (Luminal)	0.064				0.19			
Subtype (TNBC)	0.38	1.23	0.77	1.96	0.51	1.17	0.73	1.86
Subtype (HER2)	0.017	1.55	1.08	2.21	0.068	1.37	0.98	1.92

HR = Hazard Ratio; CI = Confidence Interval.

**Table 4:** Final multivariable Cox proportional-hazards models of breast cancer-specific survival including CA20 and CIN25 (based on optimal thresholds) in full models using METABRIC data. HR = Hazard Ratio; CI = Confidence Interval.

Covariates	Discovery set				Validation set			
	p-value	HR	95% CI for HR		p-value	HR	95% CI for HR	
			Lower	Upper			Lower	Upper
CA20 (high)	0.006	2.13	1.24	3.68	0.028	1.82	1.07	3.12
CIN25 (high)	0.053	1.49	0.99	2.22	0.29	1.27	0.81	1.97
Stage 0	<0.001				<0.001			
Stage I	0.001	0.48	0.31	0.75	0.005	0.53	0.34	0.82
Stage II	0.003	0.58	0.41	0.83	0.78	0.95	0.68	1.34
Stage III/IV	0.002	2.18	1.32	3.58	0.001	2.27	1.39	3.68
Chemotherapy	<0.001	2.24	1.57	3.19	0.002	1.73	1.22	2.44
Hormone therapy	0.040	1.38	1.01	1.88	0.71	0.94	0.70	1.27
Radiotherapy	0.059	0.73	0.53	1.01	0.51	1.11	0.81	1.51

**Figure 2:**
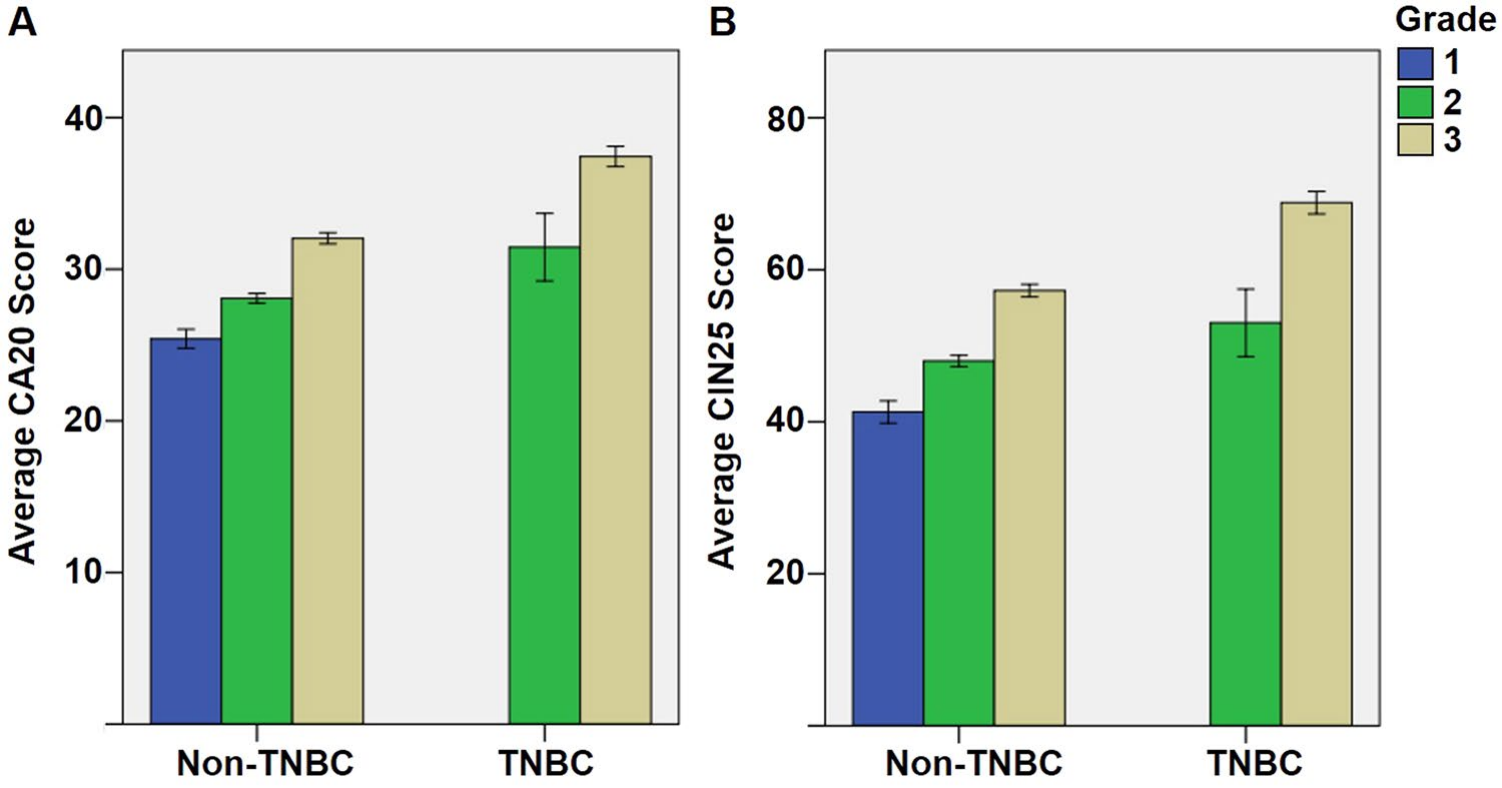
Grade-wise comparison of (**A**) average CA20 score and (**B**) average CIN25 score in non-TNBCs vs. TNBCs in the METABRIC dataset, which were significantly different at the p < 0.001 level in grade-matched comparisons. Error bars represent 95% confidence intervals.

CA20 score was highly correlated with CIN25 score (ρ = 0.93, p < 10^−6^), which may reveal that breast tumors with high CA20 scores have high levels of CIN. Although breast cancer subtype was not a common independent predictor of outcomes, we were interested to test whether CA20 and CIN25 scores differed grade-wise between TNBCs and non-TNBCs, which differ in aggressiveness. No grade 1 TNBCs were present in the dataset for comparison, but we found that average CA20 and CIN25 scores were higher in TNBCs than non-TNBCs in both grade 2 and 3 tumors per two-tailed independent samples t-tests, equal variances assumed (p < 0.001 for all) (Fig. 2), consistent with the more aggressive behavior of TNBCs compared with non-TNBCs and mirroring what we previously found for CA as assessed by microscopy^3^.

### TCGA dataset

**Figure 3:**
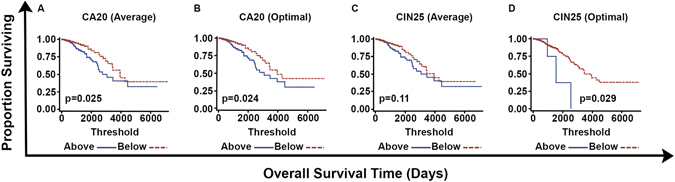
Plots of Kaplan-Meier product limit estimates of overall survival of patients in TCGA dataset stratified by (**A**) CA20 (average value), (**B**) CA20 (optimal threshold), (**C**) CIN25 (average value), and (**D**) CIN25 (optimal threshold) determined using the entire dataset.

To confirm the prognostic value of CA20 in a separate cohort, we analyzed the TCGA breast dataset. Stratification was conducted according to the average CA20 and CIN25 scores found in the entire dataset as well as optimal cutpoints in CA20 and CIN25 found in the entire dataset based on the log-rank test. In Kaplan-Meier plots, stratification into high- and low-OS groups based on CA20 (average and optimal cutpoints) was significant (p = 0.025 and p = 0.024, respectively), with high CA20 conferring a worse prognosis. For comparison, stratification by CIN25 (optimal cutpoint) was significant (p = 0.029), whereas stratification by CIN25 (average cutpoint) was not (Fig. 3). In stage-adjusted models, high CA20 scores (based on both average and optimal cutpoints) were associated with 2.72- and 2.79-fold worse OS (bootstrap-p = 0.016 and 0.008, respectively). For comparison, in stage-adjusted models, high CIN25 scores (based on both average and optimal cutpoints) were also associated with worse OS, HR = 2.31 and 4.65 (bootstrap-p = 0.026 and 0.035, respectively). However, as in the METABRIC dataset, when both CA20 and CIN25 (stratified by average or optimal cutpoints) were entered along with stage in full models, following backward variable selection (based on an α = 0.10 removal criterion), only CA20 and stage remained as predictors in full models (CA20 [stratified by average cutpoint]: HR = 2.80, p = 0.008; CA20 [stratified by optimal cutpoint]: HR = 2.55, p = 0.009), and they remained significant following bootstrapping (Table 5).

**Table 5:** Final multivariable Cox proportional-hazards models of overall survival including CA20 and CIN25 (based on average or optimal thresholds) and AJCC stage in full models using TCGA data along with p-values and 95% confidence intervals (CIs) for final model covariate hazard ratios obtained via simple bootstrapping.

Model	Covariates	HR	p-value	95% CI for HR		Bootstrap p-value	Bootstrap 95% CI for HR	
				Lower	Upper		Lower	Upper
CA20 and CIN25 (average)	CA20 (high)	2.80	0.008	1.31	5.99	0.010	1.31	7.43
	Stage III/IV	2.68	0.006	1.33	5.41	0.011	1.13	6.50
CA20 and CIN25 (optimal)	CA20 (high)	2.55	0.009	1.27	5.15	0.010	1.34	7.29
	Stage III/IV	2.87	0.008	1.32	6.25	0.012	1.14	6.16

References for hazard ratios (HRs) are CA20 (low) and stage I/II.

Although age at diagnosis was not a significant predictor of BCSS in the METABRIC dataset in final models, we recognized the possibility that it could confound analyses of OS in this independent dataset. We thus refit multivariable models entering CA20 and CIN25 (stratified by average or optimal cutpoints), AJCC stage, and age at diagnosis. In final models, CA20 remained a significant predictor, along with stage and age but not CIN25, and the hazard associated with high CA20 was even greater than in models not adjusted for age (CA20 [stratified by average cutpoint]: HR = 3.82, p = 0.001; CA20 [stratified by optimal cutpoint]: HR = 3.67, p = 0.002); furthermore, significance was retained after bootstrapping (Table 6). Because all the cases in the multivariable models were annotated with age at diagnosis, our sample size and, thus, statistical power were not diminished. Therefore, the prognostic value of CA20 adjusting for stage was upheld in this separate dataset adjusted for confounding variables, suggesting broad clinical utility for this score to predict outcomes in female breast cancer patients. Similar to our findings in the METABRIC dataset, in the TCGA dataset CA20 was very strongly correlated with CIN25 (ρ = 0.95, p < 10^−6^), suggesting that breast tumors with high CA20 scores also have high levels of CIN.

**Table 6:** Final multivariable Cox proportional-hazards models of overall survival including CA20 and CIN25 (based on average or optimal thresholds), AJCC stage, and age at diagnosis in full models using TCGA data along with p-values and 95% confidence intervals (CIs) for final model covariate hazard ratios obtained via simple bootstrapping.

Model	Covariates	HR	p-value	95% CI for HR		Bootstrap p-value	Bootstrap 95% CI for HR	
				Lower	Upper		Lower	Upper
CA20 and CIN25 (average)	CA20 (high)	3.82	0.001	1.69	8.67	0.007	1.70	13.11
	Age at diagnosis	1.04	0.010	1.01	1.07	0.028	1.00	1.08
	Stage III/IV	2.74	0.005	1.35	5.55	0.003	1.22	6.85
CA20 and CIN25 (optimal)	CA20 (high)	3.67	0.002	1.63	8.26	0.003	1.60	12.83
	Age at diagnosis	1.04	0.015	1.01	1.07	0.032	1.00	1.07
	Stage III/IV	2.50	0.011	1.24	5.05	0.009	1.14	6.05

References for hazard ratios (HRs) are CA20 (low) and stage I/II.

Finally, we were interested in exploring differences in biological processes, molecular pathways, and oncogenic signatures between CA20-high and low groups (defined by the average CA20 value), which may reveal potentially actionable biology. To this end, we performed Gene Set Enrichment Analysis (GSEA)^13^ using the TCGA dataset and explored differentially enriched biological processes, Reactome pathways, and oncogenic signatures. For the CA20-high group, 262 biological process gene sets were enriched at false discovery rate (FDR) q < 0.05 (Supplementary Table 2), but no such gene sets were significantly enriched in the CA20-low group (Supplementary Table 3). Among the most significant results, the CA20-high group was enriched in DNA repair processes, the DNA integrity checkpoint, many cell cycle processes (e.g., mitotic nuclear division, cell cycle phase transition, cell division, spindle assembly, regulation of sister chromatid segregation, mitotic spindle organization), and regulation of microtubule polymerization/depolymerization. Regarding Reactome pathways, the CA20-high group was enriched in 96 gene sets at FDR q < 0.05 (Supplementary Table 4), but the CA20-low group was not enriched in any such gene sets (Supplementary Table 5). Top enriched Reactome pathways in the CA20-high group exhibited much overlap with biological processes, including DNA repair and cell cycle pathways. For the purposes of comparison, we also compared biological processes and Reactome pathways between CIN25-high and low groups (stratified by the average CIN25 score) (Supplementary Tables 6–9), and it was found that results overlapped greatly with those from the CA20 analyses. Regarding enriched biological processes, only one gene set found in the CA20-high group (<1% of gene sets) was not found in the CIN25-high group, and only 14 gene sets found in the CIN25-high group (~5% of gene sets) were not found in the CA20-high group (Supplementary Table 10). Similarly, only four Reactome pathways enriched in the CA20-high group and four in the CIN25-high group differed (~4% of gene sets) (Supplementary Table 12). We also explored differences in oncogenic signature gene sets. We found that the CA20-high group was enriched in 13 such gene sets, including genes upregulated upon overexpression of E2F1, stimulation with sonic hedgehog (SHH) protein, and loss of retinoblastoma protein (pRb) (Supplementary Table 13), whereas the CA20-low group was not significantly enriched in any of such gene sets (Supplementary Table 13). The CIN25-high group was enriched in 12 oncogenic signature gene sets (Supplementary Table 14), all of which were found in the CA20-high group, whereas no such gene sets were significantly enriched in the CIN25-low group (Supplementary Table 15). These data suggest the CA20- and CIN25-high groupings may capture rather similar molecular tumor profiles. Finally, we tested whether the CIN25-high group was enriched in the centrosome gene ontology cellular component, and we found that it was at FDR q < 0.001 (Normalized Enrichment Score = 2.29), suggesting this group is enriched in centrosomal genes, consistent with the strong correlation we found between CA20 and CIN25 scores.

## Discussion

CA is a well-characterized hallmark of cancer^14^, especially breast cancer. Indeed, ≥75% of breast tumors (ductal carcinomas *in situ*, adenocarcinomas, invasive ductal carcinomas, or breast tumors not otherwise specified) exhibit CA^1^. Because CA promotes CIN and other aggressive phenotypes, it may be a driving force in tumorigenesis and tumor evolution that can offer insights into the clinical course of breast tumors, but only a few studies have investigated the potential prognostic value of CA. Our lab previously developed a four-gene signature, the CAI, which we demonstrated could stratify n = 162 breast cancer patients into two groups with significantly different clinical outcomes in Kaplan-Meier analysis, with high CAI based on an optimal cutpoint correlating with worse OS^3^. In the same study, we also found a non-significant trend among n = 120 breast cancer patients towards worse progression-free survival (PFS) in Kaplan-Meier analysis for tumors with high levels of CA (defined as the sum of the percentage of cells with >2 centrosomes and the percentage of cells with abnormally voluminous centrosomes based on microscopy, using an optimal cutpoint). Another study of n = 362 breast tumors found that large centrosomal size was not associated with OS or recurrence-free survival (RFS) after adjusting for tumor stage and subtype in multivariable Cox models; however, it is not known whether 2D (i.e., cross-sectional) measurements reliably estimate centrosome size, given that centrosomes are 3D structures, and numerical CA was not considered in multivariable analyses^15^. In the same study, however, Kaplan-Meier analyses revealed that high numerical CA (defined as >2 centrosomes per cell on average) was associated with worse BCSS, OS, and RFS. Thus, there is limited evidence that CA may be associated with worse outcomes in breast cancer, but it is unclear what impact CA has on survival after adjusting for potential confounders and what biological processes and pathways could be targeted therapeutically in tumors with high levels of CA.

To shed light on these questions, we developed the CA20 score based on genes encoding centrosome structural proteins and genes that have been demonstrated to induce CA following experimental perturbations in their expression. As we found for CA previously^3^, CA20 (and CIN25) were higher in the aggressive TNBC subtype than non-TNBCs in grade-matched comparisons. In analyses of two large and well-annotated breast cancer datasets (the METABRIC and TCGA breast datasets), we found that high CA20 score was associated with worse BCSS and OS after adjusting for potentially confounding factors, suggesting that CA20 could be a useful clinical tool to identify breast cancer patients at greater risk of poor outcomes. When both CA20 and CIN25 were factored into multivariable models, only CA20 was significantly associated with outcomes. This finding suggests that when CA20 is accounted for CIN25 no longer holds prognostic value. Given that we found a very strong correlation between CA20 and CIN25 in breast tumors and it has been shown by others that CA and CIN are correlated in breast tumors^15^, it is tempting to speculate that CA20 captures CIN, thus rendering CIN25 redundant, and perhaps also captures other aggressive phenotypes not encompassed by CIN25 that are consequences of CA. Given that CIN engenders karyotypic diversity within tumors, we assert that CA20 may perhaps even serve as an indirect measure of intratumor heterogeneity in breast tumors. The overlap in biological processes, Reactome pathways, and oncogenic signatures that are enriched in CA20- and CIN25-high groups is striking given that the two signatures only share one gene in common (CDK1) and suggests that they reflect relatively similar molecular tumor biology (namely, potential activation of DNA repair pathways, perhaps to cope with DNA damage occurring due to chromosome missegregation, enhanced cell cycle kinetics and microtubule dynamics, and activated E2F1 signaling), although perhaps with subtle but prognostically important qualitative and quantitative distinctions.

An exciting avenue for future research would be to test whether breast tumors with high CA20 are more susceptible to E2F1 or SHH inhibitors, drugs targeting DNA repair mechanisms (e.g., PARP inhibitors), chemotherapeutics that target the cell cycle (e.g., taxanes), or centrosome declustering drugs (such as griseofulvin, noscapinoids, PJ34, and KifC1/HSET inhibitors), which preferentially eliminate cells with CA by forcing them to construct a multipolar spindle during mitosis^16–20^. Because most normal cells do not have amplified centrosomes, declustering drugs exhibit low to no apparent toxicity to them. It will also be important to validate (through careful microscopy and rigorous quantitation) that CA20 scores indeed correlate with CA in breast tumors in future studies.

## Methods

### Dataset details and power analyses

Microarray datasets were chosen based on their availability in Oncomine^21^ and the presence of annotation regarding survival time (measured in days) and statuses and signature gene expression levels. Three microarray datasets met these criteria, including the METABRIC^22^, TCGA^23^, and Esserman^24^ breast datasets; however, power analysis suggested the Esserman dataset was too small, so it was excluded from analyses (see “Esserman dataset” below). The clinical data and log_2_ median-centered signature gene expression levels of the METABRIC and TCGA datasets were thus downloaded from Oncomine. **METABRIC dataset:** Normal breast, benign breast neoplasms, and cases without BCSS annotation were excluded from analyses, resulting in a sample size of n = 1,969 primary breast cancers. A majority of the cases were annotated for AJCC stage and whether adjuvant chemotherapy was given. The dataset was then split randomly (via random number assignment) and approximately equally into discovery and validation sets (n = 985 and n = 984, respectively; Table 1, descriptive statistics). Neither significant differences nor non-significant trends (i.e., 0.05 < p < 0.10) were found between these two sets for continuous variables (age, CA20 score, and CIN25 score; 2-tailed t-tests), ordinal variables (Nottingham grade, tumor stage, CA20 group [optimal], CA20 group [average], CIN25 group [optimal], and CIN25 group [average]; Mann-Whitney tests), or nominal variables (breast cancer subtype, chemotherapy, radiotherapy, and hormone therapy; Chi-square tests) (data not shown). **TCGA dataset:** Normal breast specimens, metastases, and male breast cancers were excluded from analyses, resulting in a sample size of n = 524 primary invasive breast cancers (Table 2, descriptive statistics). OS annotation was incomplete, so we supplemented it with clinical data downloaded from the TCGA data portal, after which all cases had OS time and status. 395 cases had AJCC stage annotation, but information about adjuvant chemotherapy was not available. We analyzed the METABRIC data to estimate whether this sample size would potentially achieve statistical power ≥0.80 in a study of the effect of CA20 on OS in stage-adjusted models with an average follow-up time of approximately 3 years, as in the TCGA study. Among METABRIC patients with invasive breast cancers (n = 1,030), the overall probability of an event (death) within 3 years was *p*_E_ = 0.12, the probability of belonging to the CA20 (optimal)-high group was *p*_H_ = 0.70, and the relative risk of death was HR = 2.34. Based on these data, it was estimated that a sample size of n = 339 would be needed to detect a HR = 2.34 with a Type I error rate of α = 0.05 and Type II error rate of β = 0.20, based on the formula to calculate the one-sided sample size in Cox proportional-hazards models^25^: $n=\frac{1}{pApBpE}{(\frac{{z}_{1-\alpha }+{z}_{1-\beta }}{\mathrm{ln}(\theta )-\mathrm{ln}({\theta }_{0})})}^{2}$. Thus, we elected not to split the data into discovery and validation sets to preserve statistical power ≥0.80 and rather implemented bootstrapping methods to more reliably estimate population parameters. **Esserman dataset:** This dataset includes n = 120 primary breast carcinomas with OS annotation and expression values for all the signature gene probes selected. Average follow up time was ~4 years, so we based power analysis on the METABRIC data 4-year OS probabilities for invasive breast cancer patients, where *p*_D_ = 0.18 and p_H_ = 0.70. Based on these criteria and using the formula as in the power analysis for the TCGA data, we estimated that n = 227 patients would be needed to detect a HR = 2.34 with a Type I error rate of α = 0.05 and a Type II error rate of β = 0.20, suggesting that the Esserman dataset would be too small for our purposes. Thus, it was excluded from analyses.

### Gene signatures and microarray probe selection

A CA gene signature was derived by searching Pubmed (September, 2015) using the search term “centrosome amplification” and filtering for experimental studies wherein manipulation of a specific gene’s expression was found to induce CA, resulting in a set of 19 genes: *AURKA* ^26, 27^, *CCNA2* ^28^, *CCND1* ^29^, *CCNE2* ^30, 31^, *CDK1* ^32^, *CEP63* ^32^, *CEP152* ^33^, *E2F1* ^34^, *E2F2* ^34^, *LMO4* ^35^, *MDM2* ^36, 37^, *MYCN* ^37^, *NDRG1* ^38^, *NEK2* ^39^, *PIN1* ^40^, *PLK1* ^41, 42^, *PLK4* ^33, 43^, *SASS6* ^44^, and *STIL* ^45^. In addition, the gene encoding the primary centrosome structural protein, *TUBG1*, was included, resulting in a set of 20 genes. The CIN25 gene signature is described in Carter *et al.* ^12^. For both datasets, many genes were represented by multiple probes. To select the probe most likely to represent the gene, probes were filtered by rational selection processes that differed by dataset since they are based on different platforms (the Illumina HumanHT-12 V3.0 R2 Array for the METABRIC dataset and the Agilent custom 244 K for the TCGA dataset). The CA20 and CIN25 scores were calculated as the sum of the normalized (log_2_ median-centered) expression levels of the signature genes. **METABRIC dataset:** Probes were filtered by preferentially selecting those targeting all isoforms (A designation). For some genes, only probes targeting some isoforms (S designation) were available. In addition, probes mapping only to the gene of interest according to a BLAST-like Alignment Tool (BLAT) search against the reference genome GRCh38 using Ensembl^46^ were preferentially selected. When multiple probes mapped to the gene of interest, the average expression level was calculated to represent that gene. **TCGA dataset:** In the absence of A and S designations, probes were filtered by performing a BLAT search as for the METABRIC data, also with averaging of normalized expression levels when multiple probes mapped to the gene of interest. Scores exhibited negative values, so for ease of interpretation, scores were converted to non-negative values by adding the minimum score value to all scores, which did not alter the results of statistical analyses.

### Survival analyses

Stratification of cases into high- and low-survival groups both by the average (as performed in the CIN25 analyses previously^12^) and by an optimal cutpoint (based on the most significant log-rank test statistic found using Cutoff Finder^47^) per the Kaplan-Meier method. Prior to fitting Cox proportional-hazards models, the proportional-hazards assumption was tested by defining each covariate as a function of time and entering this time-dependent term into a simple Cox model and determining whether there was a significant hazard in the discovery and validation sets. For no covariate was the assumption violated (data not shown). Spearman correlation (2-tailed) was performed to determine the correlation between CA20 and CIN25 scores. IBM SPSS Statistics version 21 was used for all analyses, and p < 0.05 was considered statistically significant. **METABRIC dataset:** Multivariable Cox models were fit using both discovery set data via backward-stepwise elimination of covariates (subject to an α = 0.10 removal criterion) and validation set data by entering final discovery model covariates. Full multivariable discovery model covariates included age at diagnosis (years), Nottingham grade (1, 2, or 3), AJCC stage (0, I, II, or III/IV, the latter two categories combined due to the relatively small number of stage IV cases), breast cancer subtype (luminal: ER and/or PR+, HER2−; HER2-enriched: ER/PR+/−, HER2+; triple-negative: ER/PR/HER2−), chemotherapy (yes/no), hormone therapy (yes/no), and radiotherapy (yes/no). Also, depending on the analysis, the full model either contained CA20 and CIN25 categorized based on the average score as found in the discovery set or CA20 and CIN25 categorized based on the optimal cutpoint as found in the discovery set. **TCGA dataset:** To confirm the prognostic ability of CA20 in an independent dataset, multivariable Cox models were fit using TCGA data by entering CA20 and CIN25 (average or optimal, depending on the model) and AJCC stage (categorized as I/II vs. III/IV due to relatively low numbers of stage I and IV patients) into full models (chemotherapy information was not available). Covariates were then subjected to backward-stepwise elimination (α = 0.10 removal criterion). Multivariable models were also fit including age at diagnosis (years). To more robustly estimate population parameters, the final model covariates were entered into Cox models with simple bootstrapping (1,000 iterations).

### Grade-wise comparison of average CA20 and CIN25 between TNBCs and non-TNBCs

Using the METABRIC dataset, as grade information was not available for the TCGA dataset, we compared average CA20 and average CIN25 between TNBCs and non-TNBCs grade-wise using two-tailed independent samples t-tests, guided by F-test results, and p < 0.05 was considered statistically significant.

### Gene Set Enrichment Analyses

Normalized (level 3) TCGA Hi-Seq data downloaded from the TCGA Data Portal were used for GSEA, although CA20 and CIN25 groups were specified based on average scores obtained from normalized Oncomine data. The Broad Institute GESA software version 2.2.3 was used. All 20,530 genes in the dataset were used. With the exception of not collapsing the dataset to gene symbols, all other default settings were used. Gene set databases included biological process gene ontologies (c5.bp.v5.2.symbols), Reactome pathways (c2.cp.reactome.v5.2.symbols), and oncogenic signatures (c6.all.v5.2.symbols). For the CIN25 analysis, the centrosome gene set was also used (http://amigo.geneontology.org/amigo/term/GO:0005813). FDR q < 0.05 was considered statistically significant.

## Electronic supplementary material

Supplementary Information

Supplementary Table 1

Supplementary Table 2

Supplementary Table 3

Supplementary Table 4

Supplementary Table 5

Supplementary Table 6

Supplementary Table 7

Supplementary Table 8

Supplementary Table 9

Supplementary Table 10

Supplementary Table 11

Supplementary Table 12

Supplementary Table 13

Supplementary Table 14

Supplementary Table 15

## References

[1] Chan JY (2011). A Clinical Overview of Centrosome Amplification in Human Cancers. Int. J. Biol. Sci..

[2] Godinho SA (2014). Oncogene-like induction of cellular invasion from centrosome amplification. Nature.

[3] Pannu V (2015). Rampant centrosome amplification underlies more aggressive disease course of triple negative breast cancers. Oncotarget.

[4] Ganem NJ, Godinho SA, Pellman D (2009). A Mechanism Linking Extra Centrosomes to Chromosomal Instability. Nature.

[5] Bakhoum SF, Compton DA (2012). Chromosomal instability and cancer: a complex relationship with therapeutic potential. J. Clin. Invest..

[6] Sercin O (2016). Transient PLK4 overexpression accelerates tumorigenesis in p53-deficient epidermis. Nat. Cell Biol..

[7] Chng WJ (2008). The centrosome index is a powerful prognostic marker in myeloma and identifies a cohort of patients that might benefit from aurora kinase inhibition. Blood.

[8] Chng WJ (2006). Clinical implication of centrosome amplification in plasma cell neoplasm. Blood.

[9] Pihan, G. Centrosome Dysfunction Contributes to Chromosome Instability, Chromoanagenesis, and Genome Reprograming in Cancer. *Front. Oncol.* **3**, 10.3389/fonc.2013.00277 (2013). 10.3389/fonc.2013.00277 PMC3824400 24282781

[10] Lawo S, Hasegan M, Gupta GD, Pelletier L (2012). Subdiffraction imaging of centrosomes reveals higher-order organizational features of pericentriolar material. Nat. Cell. Biol..

[11] Bauer M, Cubizolles F, Schmidt A, Nigg EA (2016). Quantitative analysis of human centrosome architecture by targeted proteomics and fluorescence imaging. EMBO J..

[12] Carter SL, Eklund AC, Kohane IS, Harris LN, Szallasi Z (2006). A signature of chromosomal instability inferred from gene expression profiles predicts clinical outcome in multiple human cancers. Nat. Genet..

[13] Subramanian A (2005). Gene set enrichment analysis: A knowledge-based approach for interpreting genome-wide expression profiles. Proc. Natl. Acad. Sci..

[14] Godinho SA, Pellman D (2014). Causes and consequences of centrosome abnormalities in cancer. Philos. Trans. R. Soc. Lond., B, Biol. Sci..

[15] Denu RA (2016). Centrosome amplification induces high grade features and is prognostic of worse outcomes in breast cancer. BMC Cancer.

[16] Ogden A (2014). Quantitative multi-parametric evaluation of centrosome declustering drugs: centrosome amplification, mitotic phenotype, cell cycle and death. Cell Death Dis..

[17] Pannu V (2014). Centrosome-declustering drugs mediate a two-pronged attack on interphase and mitosis in supercentrosomal cancer cells. Cell Death Dis..

[18] Rebacz B (2007). Identification of griseofulvin as an inhibitor of centrosomal clustering in a phenotype-based screen. Cancer Res..

[19] Castiel A (2011). A phenanthrene derived PARP inhibitor is an extra-centrosomes de-clustering agent exclusively eradicating human cancer cells. BMC Cancer.

[20] Watts CiorsdaidhA (2013). Design, Synthesis, and Biological Evaluation of an Allosteric Inhibitor of HSET that Targets Cancer Cells with Supernumerary Centrosomes. Chem. & Biol..

[21] Rhodes DR (2004). ONCOMINE: a cancer microarray database and integrated data-mining platform. Neoplasia.

[22] Curtis C (2012). The genomic and transcriptomic architecture of 2,000 breast tumours reveals novel subgroups. Nature.

[23] Comprehensive molecular portraits of human breast tumours. *Nature* **490**, 61–70 (2012). 10.1038/nature11412 PMC3465532 23000897

[24] Esserman LJ (2012). Chemotherapy response and recurrence-free survival in neoadjuvant breast cancer depends on biomarker profiles: results from the I-SPY 1 TRIAL (CALGB 150007/150012; ACRIN 6657). Breast Cancer Res. Treat..

[25] Chow S-C (2011). Sample size calculations for clinical trials. Wiley Interdisc. Rev. Comput. Stat..

[26] Zhou H (1998). Tumour amplified kinase STK15/BTAK induces centrosome amplification, aneuploidy and transformation. Nat. Genet..

[27] Meraldi P, Honda R, Nigg EA (2002). Aurora-A overexpression reveals tetraploidization as a major route to centrosome amplification in p53−/− cells. EMBO J.

[28] Hanashiro K, Kanai M, Geng Y, Sicinski P, Fukasawa K (2008). Roles of cyclins A and E in induction of centrosome amplification in p53-compromised cells. Oncogene.

[29] Nelsen CJ (2005). Short term cyclin D1 overexpression induces centrosome amplification, mitotic spindle abnormalities, and aneuploidy. J. Biol. Chem..

[30] Kawamura K (2004). Induction of Centrosome Amplification and Chromosome Instability in Human Bladder Cancer Cells by p53 Mutation and Cyclin E Overexpression. Cancer Res..

[31] Fukasawa K (2007). Oncogenes and tumour suppressors take on centrosomes. Nat. Rev. Cancer.

[32] Löffler H (2011). Cep63 Recruits Cdk1 to the Centrosome: Implications for Regulation of Mitotic Entry, Centrosome Amplification, and Genome Maintenance. Cancer Res..

[33] Dzhindzhev NS (2010). Asterless is a scaffold for the onset of centriole assembly. Nature.

[34] Lee M-Y, Moreno CS, Saavedra HI (2014). E2F Activators Signal and Maintain Centrosome Amplification in Breast Cancer Cells. Mol. Cell. Biol..

[35] Montanez-Wiscovich ME (2010). Aberrant expression of LMO4 induces centrosome amplification and mitotic spindle abnormalities in breast cancer cells. J. Pathol..

[36] Carroll PE (1999). Centrosome hyperamplification in human cancer: chromosome instability induced by p53 mutation and/or Mdm2 overexpression. Oncogene.

[37] Slack AD, Chen Z, Ludwig AD, Hicks J, Shohet JM (2007). MYCN-Directed Centrosome Amplification Requires MDM2-Mediated Suppression of p53 Activity in Neuroblastoma Cells. Cancer Res..

[38] Croessmann S (2015). NDRG1 links p53 with proliferation-mediated centrosome homeostasis and genome stability. Proc. Natl. Acad. Sci. USA.

[39] Harrison Pitner MK, Saavedra HI (2013). Cdk4 and nek2 signal binucleation and centrosome amplification in a her2+ breast cancer model. PLoS One.

[40] Suizu F, Ryo A, Wulf G, Lim J, Lu KP (2006). Pin1 Regulates Centrosome Duplication, and Its Overexpression Induces Centrosome Amplification, Chromosome Instability, and Oncogenesis. Mol. Cell. Biol..

[41] Liu X, Erikson RL (2002). Activation of Cdc2/cyclin B and inhibition of centrosome amplification in cells depleted of Plk1 by siRNA. Proc. Natl. Acad. Sci. USA.

[42] Lončarek J, Hergert P, Khodjakov A (2010). Centriole reduplication during prolonged interphase requires procentriole maturation governed by Plk1: Plk1 in procentriole maturation. Curr. Biol..

[43] Habedanck R, Stierhof YD, Wilkinson CJ, Nigg EA (2005). The Polo kinase Plk4 functions in centriole duplication. Nat. Cell Biol..

[44] Shinmura K (2015). SASS6 overexpression is associated with mitotic chromosomal abnormalities and a poor prognosis in patients with colorectal cancer. Oncol. Rep..

[45] Tang C-JC (2011). The human microcephaly protein STIL interacts with CPAP and is required for procentriole formation. EMBO J.

[46] Zerbino DR, Wilder SP, Johnson N, Juettemann T, Flicek PR (2015). The Ensembl Regulatory Build. Genome Biology.

[47] Budczies J (2012). Cutoff Finder: A Comprehensive and Straightforward Web Application Enabling Rapid Biomarker Cutoff Optimization. PLoS ONE.

