# Supplementary material for: Prognostic value of CA20, a score based on centrosome amplification-associated genes, in breast tumors
Source: Sci Rep. 2017 Mar 21;7:262. doi: 10.1038/s41598-017-00363-w (PMC5428291; doi:10.1038/s41598-017-00363-w)
Supplement: Supplementary file 1 — Supplementary Information [file 41598_2017_363_MOESM1_ESM.docx]

**Prognostic value of CA20, a score based on centrosome amplification-associated genes, in breast tumors**

Angela Ogden^1^, Padmashree C. G. Rida^1,2^, Ritu Aneja^1^*

^1^Department of Biology, Georgia State University, Atlanta, GA

^2^Novazoi Theranostics, Inc., Rolling Hills Estates, CA

***Corresponding Author**

Ritu Aneja, Department of Biology, Georgia State University, Atlanta, GA 30303; raneja@gsu.edu

**Supplementary Information**

**Supplementary Table 1.** CA20 genes, experimental manipulation that revealed a role in centrosome amplification, associated centrosomal phenotype, possible mechanism, and respective references.

**Supplementary Table 2.** Biological process (gene ontology) gene sets enriched in the CA20-high group (based on average CA20 score). Size refers to the gene set size. ES=Enrichment Score; NES=Normalized Enrichment Score; NOM=nominal; FDR=False Discovery Rate; FWER=Family-Wise Error Rate.

**Supplementary Table 3.** Biological process (gene ontology) gene sets enriched in the CA20-low group (based on average CA20 score). Size refers to the gene set size. ES=Enrichment Score; NES=Normalized Enrichment Score; NOM=nominal; FDR=False Discovery Rate; FWER=Family-Wise Error Rate.

**Supplementary Table 4.** Reactome pathway gene sets enriched in the CA20-high group (based on average CA20 score). Size refers to the gene set size. ES=Enrichment Score; NES=Normalized Enrichment Score; NOM=nominal; FDR=False Discovery Rate; FWER=Family-Wise Error Rate.

**Supplementary Table 5.** Reactome pathway gene sets enriched in the CA20-low group (based on average CA20 score). Size refers to the gene set size. ES=Enrichment Score; NES=Normalized Enrichment Score; NOM=nominal; FDR=False Discovery Rate; FWER=Family-Wise Error Rate.

**Supplementary Table 6.** Biological process (gene ontology) gene sets enriched in the CIN25-high group (based on average CIN25 score). Size refers to the gene set size. ES=Enrichment Score; NES=Normalized Enrichment Score; NOM=nominal; FDR=False Discovery Rate; FWER=Family-Wise Error Rate.

**Supplementary Table 7.** Biological process (gene ontology) gene sets enriched in the CIN25-low group (based on average CIN25 score). Size refers to the gene set size. ES=Enrichment Score; NES=Normalized Enrichment Score; NOM=nominal; FDR=False Discovery Rate; FWER=Family-Wise Error Rate.

**Supplementary Table 8.** Reactome pathway gene sets enriched in the CIN25-high group (based on average CIN25 score). Size refers to the gene set size. ES=Enrichment Score; NES=Normalized Enrichment Score; NOM=nominal; FDR=False Discovery Rate; FWER=Family-Wise Error Rate.

**Supplementary Table 9.** Reactome pathway gene sets enriched in the CIN25-low group (based on average CIN25 score). Size refers to the gene set size. ES=Enrichment Score; NES=Normalized Enrichment Score; NOM=nominal; FDR=False Discovery Rate; FWER=Family-Wise Error Rate.

**Supplementary Table 10.** Differential enrichment in biological process (gene ontology) gene sets between CA20- and CIN25-high groups (based on average CA20 and CIN25 scores).

**Supplementary Table 11.** Differential enrichment in Reactome pathway gene sets between CA20- and CIN25-high groups (based on average CA20 and CIN25 scores).

**Supplementary Table 12.** Oncogenic signature gene sets enriched in the CA20-high group (based on average CA20 score). Size refers to the gene set size. ES=Enrichment Score; NES=Normalized Enrichment Score; NOM=nominal; FDR=False Discovery Rate; FWER=Family-Wise Error Rate.

**Supplementary Table 13.** Oncogenic signature gene sets enriched in the CA20-low group (based on average CA20 score). Size refers to the gene set size. ES=Enrichment Score; NES=Normalized Enrichment Score; NOM=nominal; FDR=False Discovery Rate; FWER=Family-Wise Error Rate.

**Supplementary Table 14.** Oncogenic signature gene sets enriched in the CIN25-high group (based on average CIN25 score). Size refers to the gene set size. ES=Enrichment Score; NES=Normalized Enrichment Score; NOM=nominal; FDR=False Discovery Rate; FWER=Family-Wise Error Rate.

**Supplementary Table 15.** Oncogenic signature gene sets enriched in the CIN25-low group (based on average CIN25 score). Size refers to the gene set size. ES=Enrichment Score; NES=Normalized Enrichment Score; NOM=nominal; FDR=False Discovery Rate; FWER=Family-Wise Error Rate.
